# Transcriptomic analysis reveals that Bacillomycin D-C16 induces multiple pathways of disease resistance in cherry tomato

**DOI:** 10.1186/s12864-023-09305-5

**Published:** 2023-04-26

**Authors:** Yingying Xue, Jing Sun, Fengxia Lu, Xiaomei Bie, Yuanhong Li, Yingjian Lu, Zhaoxin Lu, Fuxing Lin

**Affiliations:** 1 College of Food Science and Technology, Nanjing Agricultural University, Nanjing, China; 2 College of Food Science and Engineering, Nanjing University of Finance and Economics, Nanjing, China; 3 School of Public Health, Xuzhou Medical University, Xuzhou, China

**Keywords:** Bacillomycin D-C16, Transcriptomic analysis, Cherry tomato, Induced resistance

## Abstract

**Background:**

Bacillomycin D-C16 can induce resistance in cherry tomato against pathogens; however, the underlying molecular mechanism is poorly understood. Here, the effect of Bacillomycin D-C16 on induction of disease resistance in cherry tomato was investigated using a transcriptomic analysis.

**Results:**

Transcriptomic analysis revealed a series of obvious enrichment pathways. Bacillomycin D-C16 induced phenylpropanoid biosynthesis pathways and activated the synthesis of defense-related metabolites including phenolic acids and lignin. Moreover, Bacillomycin D-C16 triggered a defense response through both hormone signal transduction and plant-pathogen interactions pathways, and increased the transcription of several transcription factors (e.g., AP2/ERF, WRKY and MYB). These transcription factors might contribute to the further activated the expression of defense-related genes (PR1, PR10 and CHI) and stimulated the accumulation of H_2_O_2_.

**Conclusion:**

Bacillomycin D-C16 can induce resistance in cherry tomato by activating the phenylpropanoid biosynthesis pathway, hormone signal transduction pathway and plant-pathogen interactions pathway, thus activating comprehensive defense reaction against pathogen invasion. These results provided a new insight into the bio-preservation of cherry tomato by the Bacillomycin D-C16.

**Supplementary Information:**

The online version contains supplementary material available at 10.1186/s12864-023-09305-5.

## Background

Cherry tomato fruit easily decay after harvest and during prolonged periods of storage due to humid climate and fungal diseases [1]. While synthetic fungicides have been used to control the postharvest diseases of fruits and vegetables, drawbacks including toxicity and potential hazards on human health and the environment are leading to the development of new alternatives [2]. The potential of antimicrobial peptides as novel antibiotics is widely recognized, and some serve as well-known, food-grade preservatives [3, 4]. The use of antimicrobial peptides to control plant diseases in agriculture has been proposed [5].

Cyclic lipopeptides are antimicrobial peptides that protect fruits against a number of postharvest pathogens [6, 7]. Both direct antifungal activity and induction of host defenses have been suggested as the main modes of action of cyclic lipopeptides in controlling plant disease [8]. Induction of host defense plays a crucial role in postharvest disease control and provides long-term systemic resistance to multiple pathogens. Bacillomycin D, a cyclic lipopeptide, has induced resistance to the *Rhizopus stolonifer* in cherry tomato [9]. However, the research on the molecular mechanism of Bacillomycin D inducing cherry tomato resistance is superficial, and only focuses on the research on some defense-related enzymes (such as phenylalanine ammonia lyase (PAL) and peroxidase (POD)) and the activation of some defense-related genes (such as glucanase (GLU)), chitinase (CHI), peroxidase (POD) and lipoxygenase (LOX)) [9, 10].

Transcriptomics analysis has become a valuable tool for elucidating gene expression and understanding an organism’s response to environmental stimuli. For instance, analysis using RNA-seq technology found that ABA could accelerate the ripening of tomato fruits by actively regulating genes related to the three aspects of ripening fruit, namely pigmentation, flavonoids and antioxidant capacity [11]. French et al. [12] compared the transcriptional responses of resistant and susceptible tomato roots to *Ralstonia solanacearum* infection and found that resistant tomato roots mediate resistance to *R. solanacearum* through suppression of auxin signaling and transport pathways.

In order to better understand the response to Bacillomycin D of tomato fruit, we used transcriptomics to analyze the dynamic changes in global gene expression in tomato fruits after treatment with Bacillomycin D. Through identifying differentially expressed genes (DEGs) of cherry tomato after Bacillomycin D treatment, the major metabolic pathways related to disease resistance including the phenylpropanoid biosynthesis, hormone signal transduction and plant-pathogen interactions were enriched. The transcriptional responses of key differentially expressed genes (e.g., PAL, POD and PR1) and the content of metabolic substances (e.g., phenolic acids and lignin) in the related pathways were verified by real-time PCR (RT-PCR) and HPLC.

## Results

### Transcriptome sequencing analysis of cherry tomato treated with Bacillomycin D-C16

**Fig. 1:**
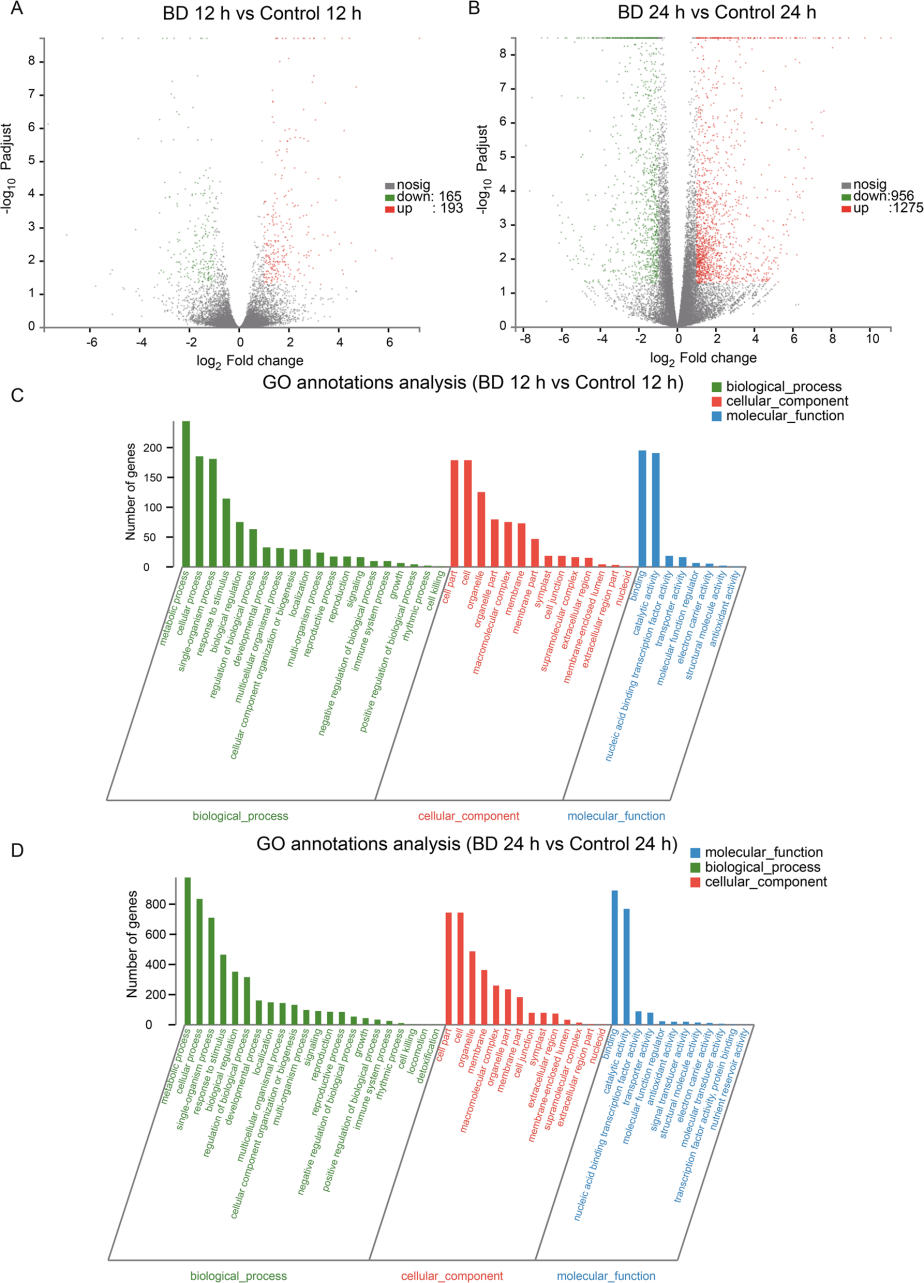
Differentially expressed genes (DEGs) represented in a volcano plot between Bacillomycin D-C16-treated (BD) and control samples after 12 h (**A**) and 24 h (**B**). GO functional classification of the DEGs after 12 h (**C**) and 24 h (**D**) of Bacillomycin D-C16 (BD) treatment. DEGs were identified between control and Bacillomycin D-C16 treatment using Wald test (adjusted *P*-value < 0.05)

To investigate the molecular responses to Bacillomycin D-C16 in cherry tomato, an RNA-Seq analysis approach was used. The transcriptome sequencing dataset which was obtained from Bacillomycin D-C16 treated and the control (Additional file 1: Table S1), showed a total of 46.30 Gb clean data. The total raw and clean reads in each sample ranged from 42,633,694 to 61,176,720 and 41,299,284 to 60,116,376, respectively. The Q20% and Q30% was higher than 98% and 95%, respectively. The GC contents were on average 43%. Two basic criteria were used to define differential gene expression: a twofold difference in transcript levels between treated and control fruits, and *P*-adjust (FDR) ≤ 0.05 (Additional file 2: Table S2, Additional file 3: Table S3). Form the results, a total of 358 DEGs were identified between the control and Bacillomycin D-C16 treatment after 12 h. With D-C16 treatment, 193 DEGs were up-regulated and 165 were down-regulated compared to the control (Fig. 1A). However, a larger number of DEGs, 2231, was found after 24 h treatment. With the longer D-C16 treatment, 1275 genes were up-regulated and 956 were down-regulated compared to the control sample (Fig. 1B). The results indicated that the plant response to Bacillomycin D-C16 treatment is fairly rapid and sustained for at least a day.

The GO enrichment analysis generated 64 and 81 GO terms between Bacillomycin D-C16 treatment and control after 12 h and 24 h, respectively (Additional file 4: Table S4, Additional file 5: Table S5). GO terms from the three sub ontologies were assigned to the DEGs: biological process, cellular component and molecular function according to the GO database (Fig. 1 C and D). In the biological process category, the highest number of DEGs were in the categories metabolic process, single-organism process, cellular process, biological regulation, response to stimulus, and regulation of biological process, may of which specific to disease resistance [13]. The most populated categories in the cellular component category were cell, organelle and membrane, cell part, which can also be related to disease resistance. Within the molecular function category, the highest number of DEGs were in the binding and catalytic activity categories, which play roles in plant hormone signal transduction.

**Fig. 2:**
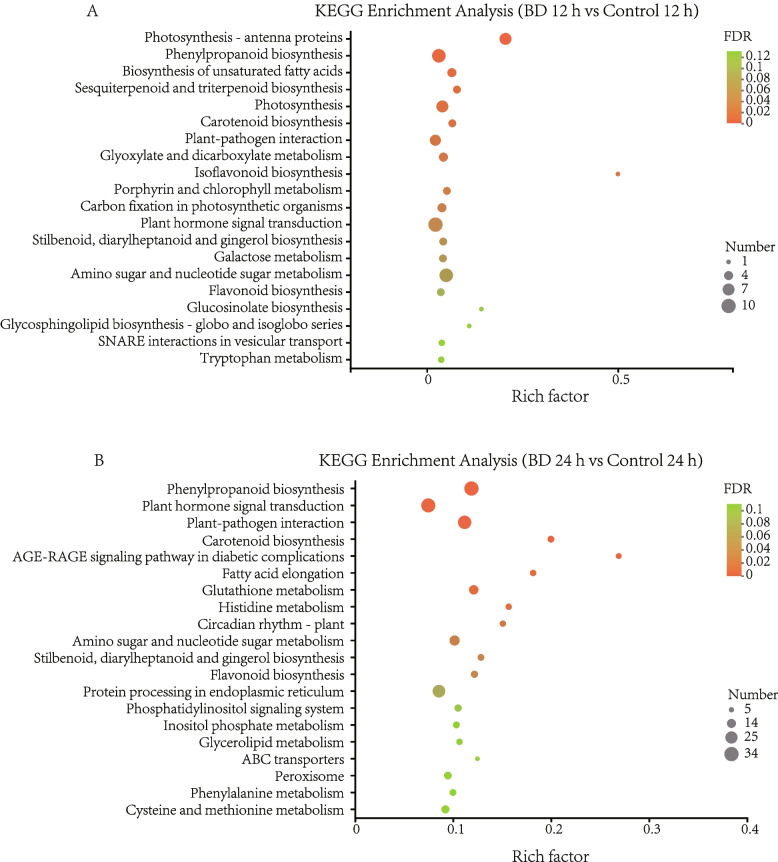
Scatter diagram of biological pathway enrichments of DEGs between Bacillomycin D-C16-treated (BD) and control samples after 12 h (**A**) and 24 h **B**. DEGs were identified between control and Bacillomycin D-C16 treatment using Wald test (adjusted *P*-value < 0.05). The rich factor is the ratio of the DEG number to the background number in a certain pathway. The greater the rich factor, the greater the degree of pathway enrichment. The size of the dots represents the number of genes, and the color of the dots represents the range of the FDR. FDR is the adjusted *P*-value ranging from 0 to 1, and a lower value indicates greater pathway enrichment

Figure 2 showed the top twenty-five KEGG pathways enriched in the Bacillomycin D-C16 treated fruit compared to the control fruit. Among these twenty-five pathways, ‘phenylpropanoid biosynthesis’ (KO 00,940), ‘plant hormone signal transduction’ (KO 04,075) and ‘plant-pathogen interactions’ (KO 04,626) contained the largest numbers of DEGs with Bacillomycin D-C16 treatment (Additional file 6: Table S6, Additional file 7: Table S7).

### Phenylpropanoid biosynthesis pathway involved in the defense of cherry tomato

**Fig. 3:**
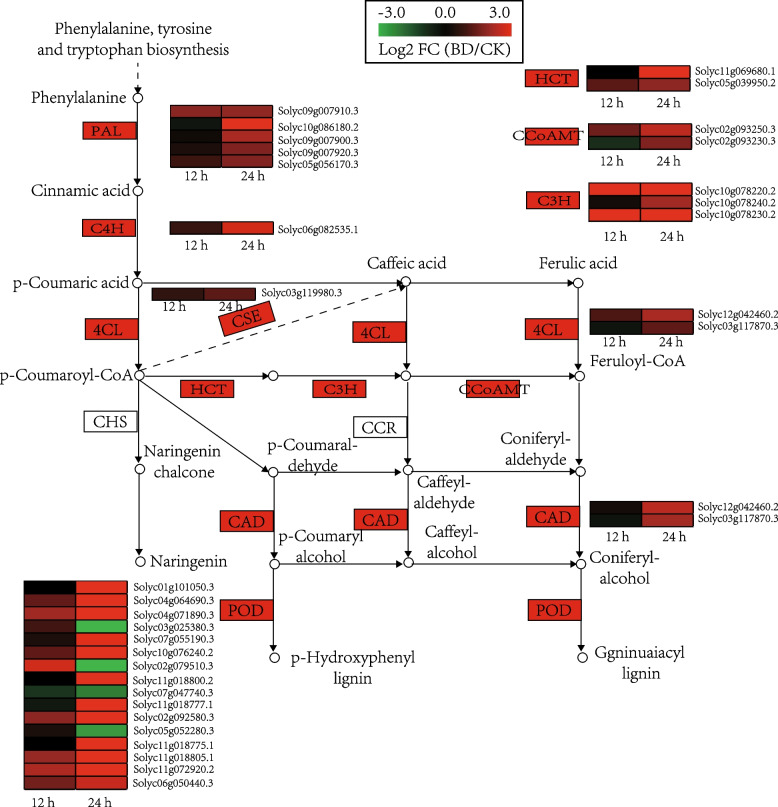
Genes that showed DEGs in response to Bacillomycin D-C16 treatment that are involved in the phenylpropanoid biosynthesis pathway. The pathway begins with the precursor amino acids and goes through to lignin derivatives. DEGs were identified between control and Bacillomycin D-C16 treatment using Wald test (adjusted *P*-value < 0.05). The color bars represent the values of log_2_ fold change (FC) between Bacillomycin D-C16-treated (BD) and control (CK) at the same time point, ranging from green (− 3) to red (3)

Phenylpropanoid biosynthesis plays a major role in plant disease resistance. At 12 h after Bacillomycin D-C16 treatment, only one gene (Solyc09g007910.3) encoding phenylalanine ammonia lyase (PAL), two genes (Solyc10g078220.2, Solyc10g078230.2) encoding coumaric acid-3-hydroxylase (C3H) and six genes (Solyc04g071890.3, Solyc02g079510.3, Solyc02g092580.3, Solyc11g018805.1, Solyc11g072920.2, Solyc06g050440.3) encoding peroxidase (POD) were up-regulated in cherry tomato (Fig. 3). At 24 h, thirty-four DEGs encoding eleven enzymes involved in the phenylpropanoid pathway were identified. In the pathway, five of the up-regulated genes (Solyc09g007910.3, Solyc10g086180.2, Solyc09g007900.3, Solyc09g007920.3, Solyc05g056170.3) encode PAL, one of the up-regulated gene (Solyc06g082535.1) encodes cinnamic acid-4-hydroxylase (C4H) and two of the up-regulated genes (Solyc12g042460.2, Solyc03g117870.3) encode 4-coumarate-CoA ligase (4CL). In addition, there were one DEG [up-regulated (Solyc03g119980.3)] encoding caffeoyl shikimate esterase (CSE), two DEGs [all up-regulated (Solyc11g069680.1, Solyc05g039950.2)] encoding quinate O-hydroxycinnamoyltransferase (HCT), three DEGs [all up-regulated (Solyc10g078220.2, Solyc10g078240.2, Solyc10g078230.2)] encoding C3H, two DEGs [all up-regulated (Solyc02g093250.3, Solyc02g093230.3)] encoding Caffeoyl-CoA O-methyltransferase (CCoAOMT), two DEGs [all up-regulated (Solyc11g011330.2, Solyc11g011340.2)] encoding cinnamonol dehydrogenase (CAD), sixteen DEGs [twelve up-regulated (Solyc01g101050.3, Solyc04g064690.3, Solyc04g071890.3, Solyc07g055190.3, Solyc10g076240.2, Solyc11g018800.2, Solyc11g018777.1, Solyc02g092580.3, Solyc11g018775.1, Solyc11g018805.1, Solyc11g072920.2, Solyc06g050440.3, Solyc06g050440.3) and four down-regulated (Solyc03g025380.3, Solyc02g079510.3, Solyc07g047740.3, Solyc05g052280.3)] encoding POD.

### Plant hormone signal transduction pathway involved in the defense of cherry tomato

**Fig. 4:**
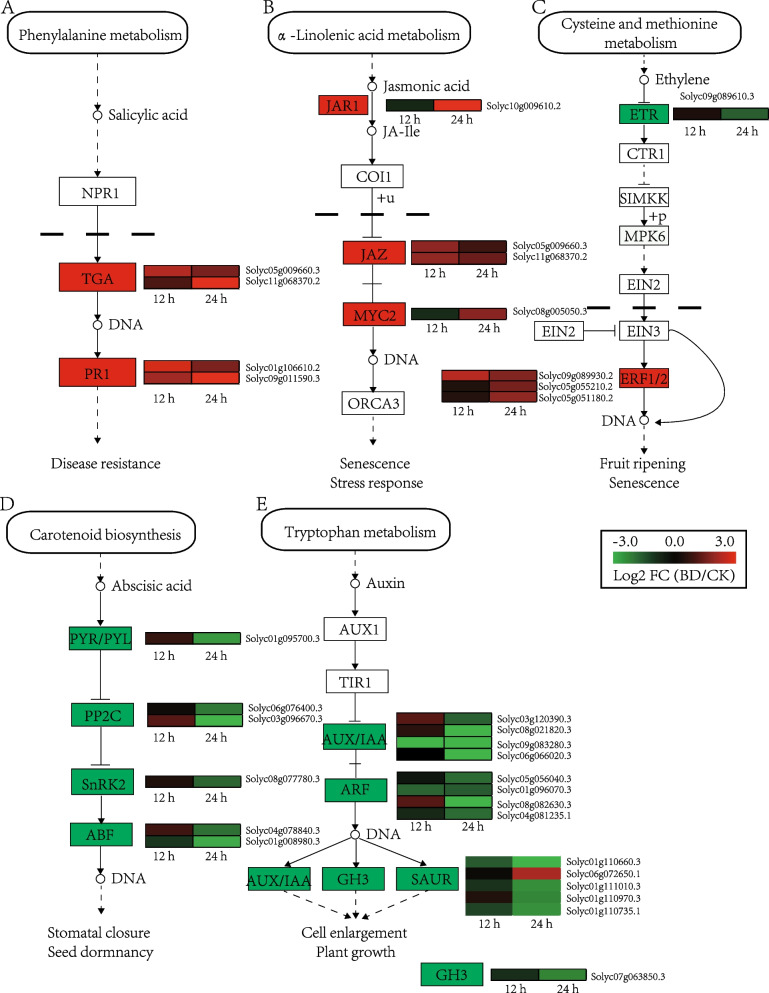
Genes that showed DEGs in response to Bacillomycin D-C16 treatment that are involved in plant hormone signal transduction pathway. **A** Salicylic acid (SA); **B** Jasmonic acid (JA); **C** Ethylene (ET); **D** Abscisic acid (ABA); **E** Auxin (AUX). DEGs were identified between control and Bacillomycin D-C16 treatment using Wald test (adjusted *P*-value < 0.05). The color bars represent the values of log_2_ fold change (FC) between Bacillomycin D-C16-treated (BD) and control (CK) at the same time point, ranging from green (− 3) to red (3)

Plant hormones are key regulators of plant disease resistance. Significant transcriptional changes in response to Bacillomycin D-C16 were observed in a group of genes involved in plant hormone signal transduction, especially those related to salicylic acid (SA), ethylene (ET), jasmonic acid (JA), abscisic acid (ABA) and auxin (AUX). In the SA-signaling pathway, two genes (Solyc05g009660.3, Solyc11g068370.2) encoding TGACG sequence-specific binding protein (TGA) and two genes (Solyc01g106610.2, Solyc09g011590.3) encoding pathogenesis-related protein 1 (PR1) were up-regulated at 12 h and 24 h (Fig. 4A). Four JA-signaling genes were identified to be associated with Bacillomycin D-C16 treatment (Fig. 4B). In the ET-signaling pathway, three genes (Solyc09g089930.2, Solyc05g055210.2, Solyc05g051180.2) encoding ethylene-responsive transcription factor 1 (ERF1) were up-regulated at 24 h (Fig. 4C). Of all ABA-signaling related genes, three genes (Solyc06g076400.3, Solyc03g096670.3, Solyc06g051940.3) encoding protein phosphatase 2C (PP2C), one gene (Solyc01g095700.3) encoding abscisic acid receptor (PYL), two genes (Solyc04g078840.3, Solyc01g008980.3) encoding ABA responsive element binding factor (ABF) and one gene (Solyc08g077780.3) encoding serine/threonine-protein kinase SRK2 (SNRK2) were greatly down-regulated by Bacillomycin D-C16 treatment at 24 h, but not unchanged at 12 h (Fig. 4D). Of the auxin-related genes, five (Solyc03g120390.3, Solyc08g021820.3, Solyc09g083280.3, Solyc06g066020.3, Solyc07g008020.3) encoding indoleacetic acid-induced protein IAA (IAA), four (Solyc05g056040.3, Solyc01g096070.3, Solyc08g082630.3,Solyc04g081235.1) encoding auxin response factor (ARF), one (Solyc07g063850.3) encoding indole-3-acetic acid-amido synthetase (GH3) and four (Solyc01g110660.3, Solyc01g111010.3, Solyc01g110970.3, Solyc01g110735.1) encoding small auxin-up RNA (SAUR) were down-regulated at 24 h (Fig. 4E).

### Calcium-mediated defense signaling pathway involved in the defense of cherry tomato

**Fig. 5:**
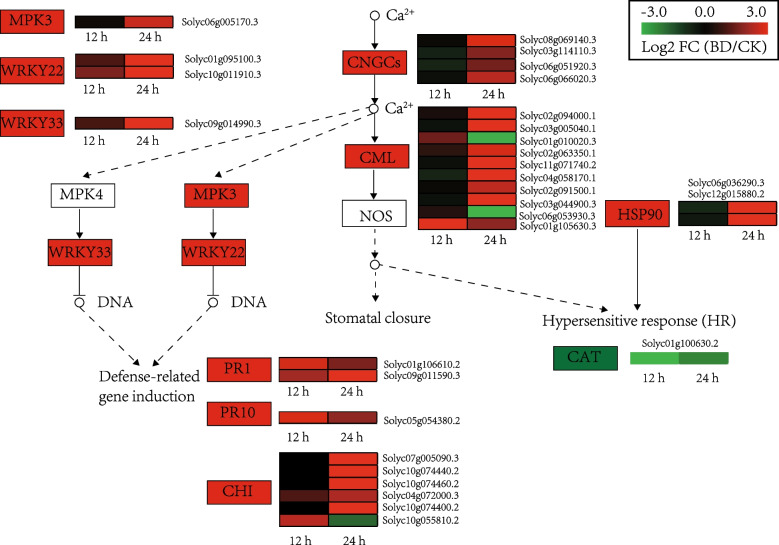
Genes that showed DEGs in response to Bacillomycin D-C16 treatment that are involved in Ca.^2+^ signal transduction pathway. DEGs were identified between control and Bacillomycin D-C16 treatment using Wald test (adjusted *P*-value < 0.05). The color bars represent the values of log_2_ fold change (FC) between Bacillomycin D-C16-treated (BD) and control (CK) at the same time point, ranging from green (− 3) to red (3)

Based on the KEGG analysis (Fig. 2), the ‘plant-pathogen interaction’ pathway was further analyzed, especially the Ca^2+^-related pathway (Fig. 5). Ca^2+^ plays an important role as a second messenger in plant cells during various developmental processes and in response to environmental stimuli [14]. Four genes (Solyc08g069140.3, Solyc03g114110.3, Solyc06g051920.3, Solyc05g050380.3) encoding cyclic nucleotide gated channel (CNGC) and seven genes (Solyc02g094000.1, Solyc03g005040.1, Solyc02g063350.1, Solyc11g071740.2,Solyc04g058170.1,Solyc02g091500.1, Solyc03g044900.3) encoding calcium-binding protein (CML) were significantly up-regulated at 24 h, which would work together to induce a hypersensitive response and cell wall reinforcement. Most DEGsencoding chitinases (CHI) were up-regulated at 24 h, while only one (Solyc10g055810.2) was up-regulated by 12 h. Additionally, a mitogen-activated protein kinase 3 (MAPK3) was up-regulated (Solyc06g005170.3) by Bacillomycin D-C16 at 24 h. Moreover, Bacillomycin D-C16 induced the defense-related genes. Two genes (Solyc01g106610.2, Solyc09g011590.3) encoding PR1, one gene (Solyc05g054380.2) encoding PR10 and one gene (Solyc04g072000.3) encoding CHI were strongly up-regulated at 12 h and 24 h. Meanwhile, Bacillomycin D-C16 induced the reactive oxygen scavenging enzyme gene. One gene (Solyc01g100630.2) encoding CAT was down-regulated at 12 h and 24 h.

### Transcription factors involved in the defense of cherry tomato

**Fig. 6:**
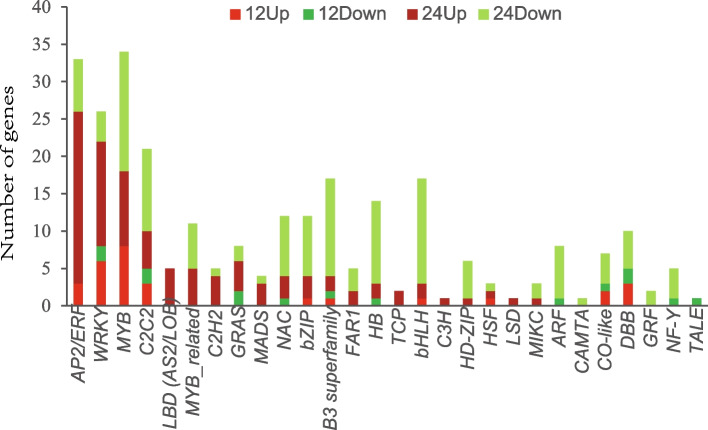
Number of genes that showed DEGs in response to Bacillomycin D-C16 treatment organized by transcription factor (TF) families. DEGs were identified between control and Bacillomycin D-C16 treatment using Wald test (adjusted *P*-value < 0.05)

Transcription factors (TFs) play important roles in regulating expression of defense genes in plant immune systems. In the present study, 274 genes encoding potential transcription factors, from 28 families, were identified among the 2589 DEGs (Additional file 8: Table S8, Additional file 9: Table S9). The major number of the differentially expressed TFs belonged to AP2/ERF, MYB, WRKY, C2H2 and B3 superfamily families (Fig. 6). Transcription factors in the AP2/ERF family were significantly up-regulated by Bacillomycin D-C16 treatment, in which two genes (Solyc07g054220.1, Solyc08g082210.3) were continuously up-regulated at two treatment time points, one gene (Solyc12g056980.1) was significantly up-regulated only at 12 h, and twenty-three genes were significantly up-regulated only at 24 h. Most MYB family genes were significantly up-regulated at 12 h and then down-regulated at 24 h, but two genes (Solyc03g005570.3, Solyc12g099140.2) were significantly up-regulated both at 12 h and 24 h. Bacillomycin D-C16 significantly increased transcription factor expression in the WRKY family, in which twenty genes were up-regulated and six genes were down-regulated. However, all transcription factors belonging to the ARF family [one gene (Solyc01g096070.3) at 12 h and seven genes (Solyc04g081235.1, Solyc03g031970.3, Solyc05g056040.3, Solyc07g016180.3, Solyc08g082630.3, Solyc11g069500.2, Solyc01g096070.3) at 24 h] were significantly down-regulated. Most B3 superfamily family genes were continuously down-regulated at two treatment time points.

### Verification of RNA-Seq date by RT-PCR

**Fig. 7:**
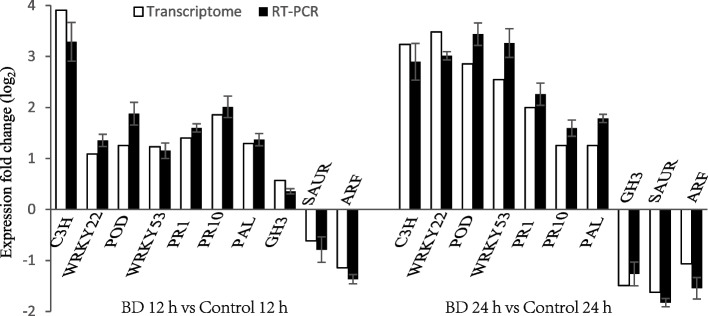
Verification of the RNA-Seq data by RT-PCR. Expression fold change of RNA-Seq and RT-PCR between Bacillomycin D-C16-treated (BD) and control samples after 12 h (left) and 24 h (right). Actin gene was used as a reference for the analysis of ten DEGs in RT-PCR. Error bars represent standard errors of 3 biological replicate samples in RT-PCR. (□) Transcriptome; (■) RT-PCR

To confirm the reliability of expression levels obtained from RNA-seq transcriptome, ten DEGs specifically involved in the defense of cherry tomato were selected (Table 1) for further investigation by RT-PCR, namely C3H (Solyc10g078230.2), WRKY22 (Solyc10g011910.3), POD (Solyc11g018805.1), WRKY53 (Solyc08g008280.3), PR1 (Solyc09g011590.3), PR10 (Solyc05g054380.2), PAL (Solyc09g007910.3), GH3 (Solyc07g063850.3), SAUR (Solyc01g111010.3), and ARF (Solyc01g096070.3) genes. The RT-PCR values of these selected genes were mostly consistent with RNA-seq results (Fig. 7). These results indicate the reliability of RNA-seq data.

**Table 1:** Primer sequence for real-time PCR

Gene function annotation	Gene ID	Revers primer (5' → 3')
C3H	Solyc10g078230.2	F: GCACCCTCCAACTCCACTAA
		R: CGCCCACACGTTAACATGTA
WRKY22	Solyc10g011910.3	F: CGAAAACAAGTGGAGCGGAA
		R: TGAATTTGTCACCGGCGAAG
WRKY53	Solyc08g008280.3	F: ACCGAGGCTCCCATAATTGT
		R: CATGCTGCTGGGTCATTCTC
GH3	Solyc07g063850.3	F: CAACCACAACCATTCCAGGG
		R: GGATTTGTCTGAGGCACGAC
PR1	Solyc09g011590.3	F: GGGTCAGCAGTGTGGAAAAG
		R:GAATTCCCAAATAAAGTGCTGCA
PR10	Solyc05g054380.2	F: ACAAGGAGATGGTGGAGCTG
		R: CCCAATGGATCCCCTTCAATC
POD	Solyc11g018805.1	F: GAACGTCGTATGGCTGCATC
		R: GCACACGATACAACTCCAGG
PAL	Solyc09g007910.3	F: GCATCCGGTGATCTTGTTCC
		R: CGAAGCCAAACCAGAACCAA
ARF	Solyc01g096070.3	F: TTGGGGTTAGACGTCTTGCT
		R: CCCACTGAGAACCCATGACT
SAUR	Solyc01g111010.3	F: GTGTCACAATTCCCTGCACT
		R: GCAACACTGTCCACTAGCAC
Actin	NM_001330119	F: AGGCACACAGGTGTTATGGT
		R: AGCAACTCGAAGCTCATTGT

### Effect of Bacillomycin D-C16 on phenylpropanoid metabolism-related enzyme activities in cherry tomato fruit

**Fig. 8:**
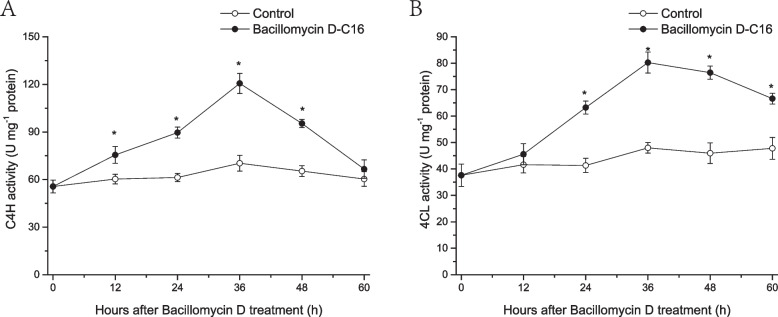
Effect of Bacillomycin D on the activities of cinnamic acid-4-hydroxylase (C4H) (**A**) and 4-coumarate-CoA ligase (4CL) (**B**) in cherry tomato fruit. Error bars represent standard errors of 3 biological replicate samples. The mark * indicates significant differences between Bacillomycin D-C16 treatment and control according to Duncan's multiple range test at *P* < 0.05

Compared with the control, Bacillomycin D-C16 triggered the activities of C4H and 4CL in cherry tomato (Fig. 8). C4H activity in cherry tomato treated with Bacillomycin D-C16 rapidly increased, reached a peak value at 36 h, and then decreased from 36 to 60 h (Fig. 8A). Further comparison demonstrated Bacillomycin D-C16-treated fruit had significantly higher C4H activity (*P* < 0.05) than control treatment from 12 to 48 h.

4CL activity increased gradually until 36 h after Bacillomycin D-C16 treatment as compared to the activity in control fruit, and then decreased. Bacillomycin D-C16 showed 67.1% higher 4CL activity than control treatment at 36 h (Fig. 8B).

### Effect of Bacillomycin D-C16 on phenolic acid and lignin contents in cherry tomato fruit

**Fig. 9:**
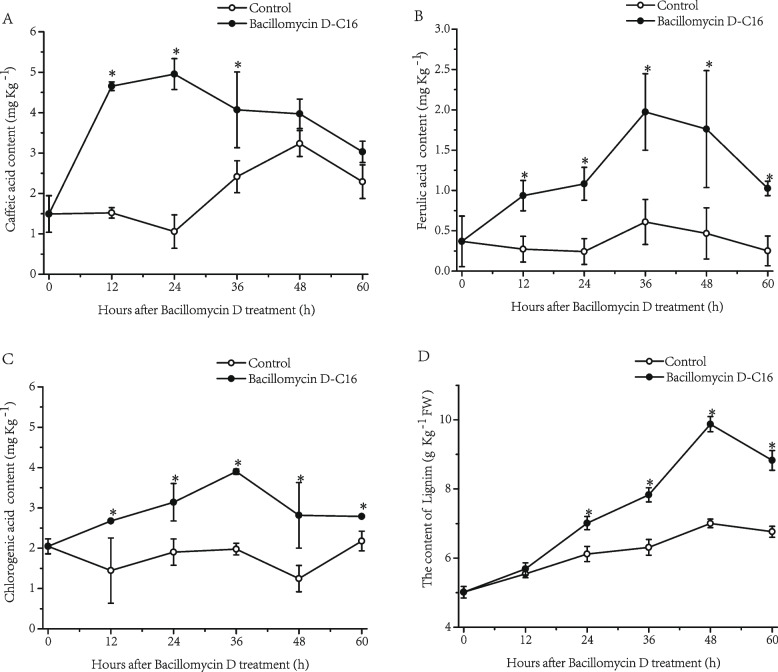
Effect of Bacillomycin D-C16 on the contents of caffeic acid (**A**), ferulic acid (**B**), chlorogenic acid (**C**) and lignin (**D**) in cherry tomato fruit. Error bars represent standard errors of 3 biological replicate samples. The mark * indicates significant differences between Bacillomycin D-C16 treatment and control according to Duncan's multiple range test at *P* < 0.05

The activation of key genes in the phenylpropanoid biosynthesis pathway could lead to the synthesis and accumulation of disease resistance-related substances, like phenolic acids and lignin [15]. Bacillomycin D-C16 treatment altered the levels of phenolic acids over 60 h (Fig. 9). Even with wounding, the caffeic acid content of the control fruit did not change significantly within 24 h, but then increased over twofold from 24 to 48 h, and declined slightly by 60 h (Fig. 9A). In the wounded fruit also treated with Bacillomycin D-C16, caffeic acid exhibited a sharp increase at 12 h, a slight increase from 12 to 24 h, and then a gradual reduction from 24 to 60 h. The fruit treated with Bacillomycin D-C16 had a remarkably higher (*P* < 0.05) caffeic acid content than control treatment from 12 to 36 h.

The content of ferulic acid showed no significant change in the control from 12 to 60 h (Fig. 9B). In contrast, the ferulic acid content in Bacillomycin D-C16-treated fruit increased, reaching its peak value 36 h after treatment, and then slightly decreased. Bacillomycin D-C16 significantly increased ferulic acid content in cherry tomato during the whole storage period (*P* < 0.05).

The content of chlorogenic acid also did not change significantly in the control fruit within 36 h, but showed a slight decreased from 36 to 48 h, and a return to its initial levels by 60 h. On the other hand, chlorogenic acid did increase steadily through the first 36 h after Bacillomycin D-C16 treatment, and then showed a slight reduction from 48 to 60 h. Further comparison indicated that the chlorogenic acid content of Bacillomycin D-C16 treatment was significantly (*P* < 0.05) higher from 12 to 60 h when compared with the control group.

The lignin content increased gradually until 48 h after Bacillomycin D-C16 treatment as compared to the lignin content in control, and showed a sharp reduction from 48 to 60 h. Bacillomycin D-C16-treatment resulted in 142% higher lignin content compared to the control treatment at 48 h (Fig. 9D).

### Effect of Bacillomycin D-C16 on ROS scavenging enzyme activity and H_2_O_2_ content in cherry tomato fruit

**Fig. 10:**
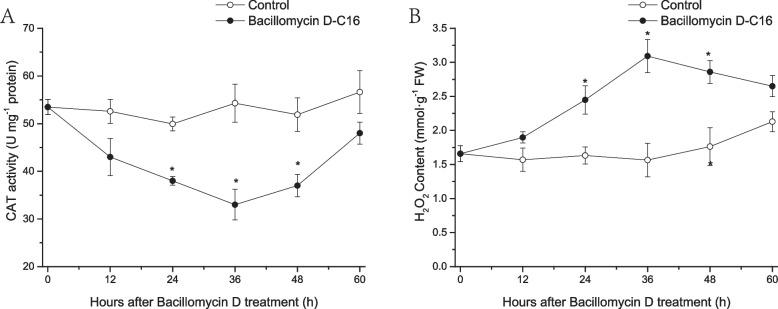
Effect of Bacillomycin D on the activity of catalase (CAT) (**A**) and the content of H_2_O_2_ (**B**) in cherry tomato fruit. Error bars represent standard errors of 3 biological replicate samples. The mark * indicates significant differences between Bacillomycin D-C16 treatment and control according to Duncan's multiple range test at *P* < 0.05

Activation of key genes in the plant-pathogen interactions pathway could lead to initiation of related kinasekinase hypersensitive response (HR), such as increased ROS [16]. CAT is an active oxygen scavenging enzyme that can reduce H_2_O_2_ to H_2_O [17]. CAT activity in the treatment group was significantly lower (*P* < 0.05) than in the control group from 24 to 48 h, with a peak at 36 h (Fig. 10A).

H_2_O_2_ content increased significantly (*P* < 0.05) in cherry tomato treated with Bacillomycin D-C16 from 24 to 48 h (Fig. 10B). H_2_O_2_ content in Bacillomycin D-C16 treated fruit was 50.2%, 75.6% and 62.5% higher than in control fruit at 24, 36 and 48 h after treatment, respectively.

## Discussion and conclusion

The induction of host defense can become a long-term systemic resistance to a variety of pathogens that can be especially effective in controlling postharvest disease. Bacillomycin D, a cyclic lipopeptide, can induce the resistance to soft rot disease in cherry tomato fruit. However, knowledge of the process underlying Bacillomycin D induction of disease resistance in fruit is limited to single enzymes (such as GLU, CHI, POD and LOX), or genes (GLU, CHI, PR1 and NPR1) [9]. In the present work, we used Illumina sequencing technology to further study the molecular mechanism of Bacillomycin D-induced tomato fruit resistance. The transcriptomics identified a total of 358 and 2231 DEGs in pairwise comparisons of the control and Bacillomycin D-C16-treated fruit at 12 h and 24 h, respectively (Fig. 1). GO categorization of the DEGs revealed that metabolic process, cellular processes, single organism processes, response to stimulus, cell, cell part, organelle, catalytic activity and binding were the most differentially regulated processes occurring during induction of resistance within the tomato fruit. KEGG enrichment analysis revealed that the highest DEG representation were in pathways for ‘phenylpropanoid biosynthesis’, followed by those for ‘plant hormone signal transduction’ and ‘plant-pathogen interaction’.

The phenylpropanoid pathway plays a significant role in the response to biological and abiotic stresses [15]. Phenylalanine is the final product of the plant oxalate pathway, which is the center for the biosynthesis of phenylpropanoids [18]. L-phenylalanine is converted to trans-cinnamic acid by PAL, and then C4H catalyzes trans-cinnamic acid to hydroxycinnamic acid, which is used to synthesize caffeic acid and ferulic acid. 4CL catalyzes the synthesis of CoA thioesters of these acids, which are finally used for the biosynthesis of flavonoids. At the same time, in the presence of CAD and POD, the CoA derivatives of these acids are catalyzed to lignin [19]. In this study, the results on the transcriptomics showed that the expression levels of PAL and POD were up-regulated in Bacillomycin D-C16 treatment. This was similar to an earlier report on wheat that showed that surfactin enhanced the levels of POD and PAL transcripts [20]. In addition, the expression levels of C4H, C3H, 4CL, CCoAOMT and CAD, other crucial genes related to phenylpropanoid biosynthesis, were also up-regulated with Bacillomycin D-C16 treatment. Chlorogenic acid, caffeic acid and ferulic acid are three important phenolic substances and intermediates of the phenylpropanoid biosynthesis pathway [21]. Lignin, the product of phenolic acid-derived compounds, gives structural support and pathogen defense for the fruit [22]. To confirm up-regulated genes in the phenylpropanoid pathway at biochemical level, the activities of key enzymes and the concentrations of four metabolites in cherry tomato were determined. Our previous study has found that Bacillomycin D-C16 could activate the activities of phenylpropanoid metabolism-related enzymes (PAL and POD) [9]. Results from this study showed that Bacillomycin D-C16 induced significantly higher activities of phenylpropanoid metabolism-related enzymes (C4H and 4CL) and stimulated phenolic acid and lignin accumulation (Figs. 8 and 9). Similar results can be observed in previous studies which reported that the accumulation of phenolic acid and lignin in postharvest fruit may result from increased activities of phenylpropanoid metabolism-related enzymes [15, 23].

Six phytohormones—salicylic acid (SA), jasmonic acid (JA), ethylene (ET), auxin (IAA) and abscisic acid (ABA)—are known to play vital roles in regulating the defense response of plants to various pathogens and abiotic stresses [24]. Salicylic acid interacts with downstream NPR1 to activate a great quantity of WRKY genes, initiating transcriptional expression of various disease resistance genes [25]. JA and ethylene regulate the resistance of plants to necrotic pathogens by activating the MYC response branch and the ERF response branch, respectively [26]. Auxin could negatively affect plant defense capabilities by interfering with other hormone signaling pathways or with PTI [27]. Abscisic acid can on one hand positively regulate plant defense responses by the synergistic enhancement of the MYC branch in the JA response pathway, and on the other hand can negatively regulate plant defense responses through inhibition of the SA defense responses [26]. Previous studies have revealed that cyclic lipopeptide iturin A induces the defense response of Arabidopsis by activating SA and JA signaling pathways [28]. The expression of PR1 and PDF1.2 genes, involved in the SA- and JA-dependent defense signaling pathways in *Arabidopsis*, were enhanced in *Bacillus amyloliquefaciens* FZB42 containing cyclic lipopeptide; in contrast their expression levels showed no significant changes in FZB42DsfpDalsS without the cyclic lipopeptide [29]. In rice, fengycin and mycosubtilin up-regulated the expression of key genes in the SA, JA, ET, and Auxin pathways to enhance the defense response to pathogens [30]. Similarly, Bacillomycin D-C16 could significantly up-regulate the expression of key genes, like TGA and PR1 in the SA pathway, JAZ, JZR and MYC in the JA pathway, and ERF in the ET pathway. However, the expression of IAA, ARF, GH3 and SAUR genes in the Auxin pathway were down-regulated after treatment with Bacillomycin D-C16, which is similar to the results of Ding et al. [31]. In addition, we also found that Bacillomycin D-C16 could remarkably down-regulate the expression of the PYL, PP2C, ABF and SNRK2 genes in the ABA pathway. Therefore, the above results indicated that Bacillomycin D-C16 could regulate the expression of genes in the signal transduction pathways of several plant hormones, thereby indirectly affecting the defense response of fruits.

The number of DEGs related to the “plant-pathogen interactions” pathway was higher with Bacillomycin D-C16 treatment compared to control. We analyzed this subset of DEGs, especially those associated with the Ca^2+^ signaling pathway. Calcium signaling has been implicated in plant responses to a range of biotic and abiotic stresses [32]. Under external stimulation, cyclic nucleotide gated channels are activated, leading to Ca^2+^ influx. Cytosolic Ca^2+^ elevation leads to an increase amount of Ca^2+^ complexed with calmodulin (or CML), which induces a hypersensitive response [33]. The plant defenses activated by cyclic lipopeptides is closely related to Ca^2+^ influx [34, 35]. In this study, four DEGs (all up-regulated) encoding CNGC, ten DEGs encoding CML (eight up-regulated and two down-regulated) and one DEGs encoding CALM (up-regulated), were detected in Bacillomycin D-C16-treated cherry tomato. This result strongly indicated that Ca^2+^-related genes are involved in modulating these defense responses. In addition, one protein kinase gene (MPK3 and defense-related genes (including two PR1 genes, one PR10 gene and three CHI genes), known to be downstream of Ca^2+^ signaling, were more highly expressed after Bacillomycin D-C16 treatment. However, one reactive oxygen species scavenging enzyme gene (CAT) in the downstream of Ca^2+^ signaling was down-regulated after treatment with Bacillomycin D-C16, which is similar to the results of Hou et al. [36]. To validate the differential genes in plant-pathogen interactions pathway at biochemical level, the activities of key enzymes (CHI and CAT) and the content of H_2_O_2_ in cherry tomato were measured. Our previous study has found that Bacillomycin D-C16 could activate the activity of defense-related enzyme (CHI) [9]. Results from the present study demonstrated that Bacillomycin D-C16 induced significantly lower activity of CAT and stimulated H_2_O_2_ accumulation in cherry tomato (Fig. 10). These results reveal that Bacillomycin D-C16 increased transcript levels of key genes in the plant-pathogen interactions pathway, which could lead to the activation of defense-related genes and enzymes and the accumulation of H_2_O_2_ in cherry tomato.

Transcription factors, such as AP2/ERF, NAC, MYB, and WRKY, play crucial roles in plant defense responses [37]. AP2/ERF and WRKY genes could respond defensively to environmental stimuli through regulating the signal transduction pathway [38, 39]. These genes were over-expressed in this dataset, as 33 AP2/ERF genes and 26 WRKY genes were determined exhibiting differential expression among a total of 2589 DEGs.

**Fig. 11:**
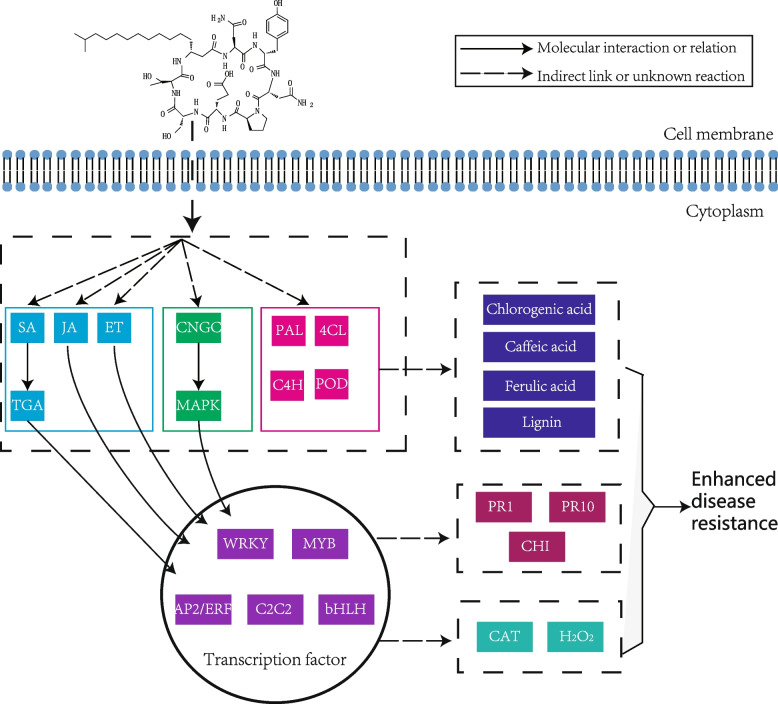
Proposed network of Bacillomycin D-C16-induced defense responses in cherry tomato.  represents molecular interaction or relation  represents indirect link or unknown reaction.

Based on the above research results, a probable molecular network for the defense response of cherry tomato to Bacillomycin D-C16 treatment is proposed (Fig. 11). Briefly, Bacillomycin D-C16 inserts into the cell membrane and simultaneously triggers relevant transduction pathways. Whether directly or indirectly, plant hormone and calcium signal transduction pathways and phenylpropanoid biosynthesis are activated. Plant hormones and calcium pathways further activate TFs, which activate the expression of downstream defense-related genes (PR1, PR10 and CHI) and stimulate the accumulation of H_2_O_2_. The phenylpropanoid biosynthesis pathway promotes the accumulation of defense-related substances, like phenolic acids and lignin. The expression of resistance-related genes and the accumulation of resistance-related substances together enhance the disease resistance of cherry tomato.

In conclusion, Bacillomycin D-C16 can induce resistance in cherry tomato by activating the phenylpropanoid biosynthesis pathway, hormone signal transduction pathway and plant-pathogen interactions pathway, thus activating comprehensive defense reaction against pathogen invasion. This study is the first step in understanding the molecular mechanism of Bacillomycin D induced resistance in cherry tomato. The transcriptome data generated here will help guide further research to develop new strategies to manage cherry tomato disease. Future work can use other omics techniques to analyze the complex molecular mechanism of Bacillomycin D inducing resistance to cherry tomato fruit.

## Materials and methods

### Fruit material and Bacillomycin D-C16

Fruit from the cherry tomato cultivar Qian xi were harvested at mature pink stages from a greenhouse located in Nanjing, Jiangsu Province, China. Fruit were immersed in 0.1% sodium hypochlorite solution for 2 min, rinsed using distilled water, and dried at room temperature.

Bacillomycin D-C16 was purified using high performance liquid chromatography (HPLC) according to Lin et al. [40].

### Fruit treatment

One wound (5 mm diameter and 2 mm deep) was made at the equator of the cherry tomato with a sterile borer. 50 μL of Bacillomycin D-C16 (50 mg L^−1^) or sterile distilled water (as control) was added to the wound. At 0, 12, 24, 36, 48 and 60 h after treatment, wounded tissues of 100 cherry tomatoes were taken with a sterile borer (15 mm diameter and 5 mm deep) at each treatment time point [9], immediately frozen in liquid nitrogen, then stored at -80 °C.

### RNA extraction and Illumina sequencing

Cherry tomato samples after 12 h and 24 h of treatment were used for this experiment. Total RNA was extracted from the frozen tissue using Plant RNA Purification Reagent (Invitrogen, Carlsbad, USA). The total RNA quality was evaluated by a 2100 Bioanalyser (Agilent, Santa Clara, USA) and quantified using the NanoDrop-2000. The RNA-seq transcriptome library was prepared following TruSeq™ RNA Sample Preparation Kit (Illumina, San Diego, USA). In brief, mRNA was isolated from 5 μg of total RNA using polyA selection by oligo (dT) beads and then fragmented by fragmentation buffer. First-strand cDNA was synthesized with mRNA as template, followed by second-strand synthesis to form stable double-stranded structure. Second-strand cDNA was enrichment by PCR, and a 200–300 bp target band was recovered using 2% agarose gel. After quantification with TBS380 (Turner Biosystems, Sunnyvale, USA), paired-end RNA-seq sequencing library was sequenced with the Illumina NovaSeq 6000 sequencer (2 × 150 bp read length). The raw paired end reads were trimmed and quality controlled by fastp (https://github.com/OpenGene/fastp) with default parameters. Then clean reads were separately aligned to reference genome (ITAG3.2) with orientation mode using HISAT2 (version 2.1.0, http://ccb.jhu.edu/software/hisat2/index.shtml) [41]. All RNA-seq analyses were performed in three biological replicates.

To identify differentially expressed genes (DEGs) between Bacillomycin D-C16 treatment and control, the expression level of each transcript was calculated according to Fragments Per kb Per Million Reads (FPKM) method. RSEM (version 1.2.31, http://deweylab.biostat.wisc.edu/rsem/) was utilized for quantify gene abundances [42]. Differential expression analysis was performed using the DESeq2 (version 1.10.1), DEGs with |log_2_ fold change (FC)|≥ 1 and P-adjust ≤ 0.05 were considered to be significantly different expressed genes software [43]. Furthermore, functional-enrichment analysis including GO (Gene Ontology, http://www.geneontology.org) and KEGG (Kyoto Encyclopedia of Genes and Genomes, http://www.genome.jp/kegg/) were performed to identify which DEGs were significantly enriched in GO terms and metabolic pathways at *P*-adjust (FDR) ≤ 0.05 compared with the whole-transcriptome background. GO functional enrichment and KEGG pathway analysis were carried out by Goatools (version 0.6.5, https://github.com/tanghaibao/Goatools) and KOBAS (version 2.1.1, http://kobas.cbi.pku.edu.cn/home.do) [44].

### RT-PCR analysis

Total RNA after 12 h and 24 h of treatment were used for this experiment. For each sample, total cDNAs were synthesized using 4 μL 5 × HiScript II qRT SuperMix, 1 μL total RNA and 15 μL RNase-free ddH_2_O at 50 ºC for 15 min and 85 ºC for 5 s. The resulting cDNA was used as the template for subsequent RT-PCR amplification. RT-PCR experiments were carried out using SYBR Green Kit (Vazyme Biotech, Jiangsu, China). Actin gene was used as a reference for the analysis of ten differentially expressed genes: C3H, WRKY22, POD, WRKY53, PR1, PR10, PAL, GH3, SAUR and ARF. Sequences of primers used were listed in Table 1. Assays were conducted using Applied Biosystems (ABI) Step One Plus (Applied Biosystems, Waltham, USA). Relative quantifications were calculated using the 2^−△△C(T)^ method [45]. All RT-PCR analyses were performed in three biological replicates.

### Determination of phenylpropanoid metabolism-related enzymes

4CL and C4H activities were determined according to Wei et al. [46]. The enzyme activity was expressed as U mg^−1^ protein. One unit of 4CL and C4H activity was defined as a 0.01 decrease at OD_333_ and OD_340_ per min.

### Determination of phenolic acids and lignin content

Phenolic acids were analyzed according to Sgherri et al. [47]. Wounded tissues (1 g) were homogenized with 50% methanol containing 1% HCl. The homogenates were ultrasonicated for 60 min and then centrifuged at 12 000 × g and 4 °C. After that, the supernatant was collected, and the extraction was repeated again twice on the pellet. The methanolic extracts were vacuum-dried, resuspended in 1.5 mL of methanol, and filtered through a membrane filter (0.22 µm) for HPLC analysis.

Phenolic acids were measured by high performance liquid chromatography (UltiMate 3000, Dionex, California, USA) with a 250 mm × 4.6 mm ZORBAX SB-Aq C18 column (Agilent, California, USA). The mobile phase consisted of solvent A (2% acetic acid) and solvent B (acetonitrile). A 20 μL aliquot of sample was loaded and separated on the column by a linear biphasic elution gradient of 100–85% solvent A over 30 min, 85–50% A over 50 min, 50–0% A over 55 min, at a flow rate of 0.6 mL min^−1^. Elution was monitored by UV detection at 280 nm. The concentration of phenolic acids was expressed as mg Kg^−1^. Each experiment was conducted in triplicate.

Lignin content was measured according to Dong et al. [48]. Sample (1 g) was homogenized with 95% ethanol and then centrifuged at 12 000 × g and 4 °C. The precipitate was collected, and incubated with 1 mL 25% bromized acetyl-acetic acid for 40 min at 70 °C. After that, 2 mL acetic acid and 0.1 mL 7.5 mol L^−1^ hydroxylamine hydrochloric acid were added and centrifuged. The absorbance of the supernatant was determined at 280 nm. Lignin compounds expressed as g Kg^−1^ fresh weight. Each experiment was conducted in triplicate.

### Determination of ROS scavenging enzyme and H_2_O_2_ content

CAT activity was assayed as described by Imahori et al. [49]. One unit of CAT activity was defined as the amount of enzyme that caused a change of 0.01 in absorbance in one minute at 240 nm.

H_2_O_2_ content was determined with the H_2_O_2_ assay kit (Solarbio, Beijing, China) according to the protocol of manufacturer. The absorbance of samples at 415 nm was used to determine H_2_O_2_ content. 

### Data analysis

The data are expressed as mean ± standard deviation. All statistical analysis was analyzed by SPSS software (SPSS Institute, Chicago, USA). *P* < 0.05 was considered significant.

### Supplementary Information

**Additional file 1: Table S1.** Overview of the transcriptome sequencing dataset and quality check.

**Additional file 2: Table S2.** Table of RNA-seq data after Bacillomycin D-C16 treatment versus control at time 12 h.

**Additional file 3: Table S3.** Table of RNA-seq data after Bacillomycin D-C16 treatment versus control at time 24 h.

**Additional file 4: Table S4.** Table of GO enrichment analysis after Bacillomycin D-C16 treatment versus control at time 12 h.

**Additional file 5: Table S5.** Table of GO enrichment analysis after Bacillomycin D-C16 treatment versus control at time 24 h.

**Additional file 6: Table S6.** Table of KEGG enrichment analysis after Bacillomycin D-C16 treatment versus control at time 12 h.

**Additional file 7: Table S7.** Table of KEGG enrichment analysis after Bacillomycin D-C16 treatment versus control at time 24 h.

**Additional file 8: Table S8.** Table of transcription factors after Bacillomycin D-C16 treatment versus control at time 12 h.

**Additional file 9: Table S9.** Table of transcription factors after Bacillomycin D-C16 treatment versus control at time 24 h.

## Data Availability

The RNA-Seq data have been deposited in NCBI (BioProject: PRJNA842836). https://www.ncbi.nlm.nih.gov/bioproject/PRJNA842836.
