# Correction: Transcriptomic analysis reveals that bacillomycin D-C16 induces multiple pathways of disease resistance in cherry tomato

**DOI:** 10.1186/s12864-023-09549-1

**Published:** 2023-08-10

**Authors:** Yingying Xue, Jing Sun, Fengxia Lu, Xiaomei Bie, Yuanhong Li, Yingjian Lu, Zhaoxin Lu, Fuxing Lin

**Affiliations:** 1 College of Food Science and Technology, Nanjing Agricultural University, Nanjing, China; 2 College of Food Science and Engineering, Nanjing University of Finance and Economics, Nanjing, China; 3 School of Public Health, Xuzhou Medical University, Xuzhou, China

**Correction: BMC Genomics 24, 218 (2023)**

10.1186/s12864-023-09305-5

Following publication of the original article [1], it was reported that there was an error in the presentation of Jing Sun, Yuanhong Li and Yingjian Lu’s affiliations.

**Jing Sun’s** correct unit of affiliation is **College of Food Science and Engineering, Nanjing University of Finance and Economics, Nanjing, China**.

**Yuanhong Li’s** correct unit of affiliation is **School of Public Health, Xuzhou Medical University, Xuzhou, China**.

**Yingjian Lu’s** correct unit of affiliation is **College of Food Science and Engineering, Nanjing University of Finance and Economics, Nanjing, China**.

The correct author affiliations are given in this Correction article and the original article [1] has been updated.
