# Supplementary material for: Transcriptomic analysis reveals that Bacillomycin D-C16 induces multiple pathways of disease resistance in cherry tomato
Source: BMC Genomics. 2023 Apr 26;24:218. doi: 10.1186/s12864-023-09305-5 (PMC10131338; doi:10.1186/s12864-023-09305-5)
Supplement: Supplementary file 1 — Additional file 1: Table S1. Overview of the transcriptome sequencing dataset and quality check. [file 12864_2023_9305_MOESM1_ESM.docx]

Table S1 Overview of the transcriptome sequencing dataset and quality check

| Samples | Raw reads | Clean reads | Q20(%) | Q30(%) | GC content(%) |
| --- | --- | --- | --- | --- | --- |
| CK12h_1 | 59024648 | 58288614 | 98.81 | 95.82 | 42.71 |
| CK12h_2 | 60535858 | 59680288 | 98.63 | 95.38 | 42.74 |
| CK12h_3 | 57345438 | 56593732 | 98.73 | 95.65 | 43.02 |
| CK24h_1 | 61176720 | 60116376 | 98.73 | 95.67 | 42.95 |
| CK24h_2 | 49636760 | 48634246 | 98.69 | 95.56 | 43.24 |
| CK24h_3 | 55315752 | 54455694 | 98.7 | 95.6 | 43.08 |
| BD12h_1 | 42633694 | 41299284 | 98.53 | 95.22 | 42.51 |
| BD12h_2 | 60601866 | 59214074 | 98.56 | 95.23 | 42.81 |
| BD12h_3 | 59097932 | 57602396 | 98.62 | 95.42 | 42.67 |
| BD24h_1 | 50272762 | 49675098 | 98.66 | 95.44 | 43.4 |
| BD24h_2 | 59220192 | 58431808 | 98.65 | 95.45 | 43.51 |
| BD24h_3 | 45687732 | 45102238 | 98.58 | 95.22 | 43.32 |
